# Role of the Ubiquitin System in Stress Granule Metabolism

**DOI:** 10.3390/ijms23073624

**Published:** 2022-03-26

**Authors:** Nazife Tolay, Alexander Buchberger

**Affiliations:** Department of Biochemistry, Biocenter, University of Würzburg, 97074 Würzburg, Germany; nazife.tolay@uni-wuerzburg.de

**Keywords:** 26S proteasome, p97/VCP, Cdc48, DUB, G3BP, granulostasis, granulophagy, ALS

## Abstract

Eukaryotic cells react to various stress conditions with the rapid formation of membrane-less organelles called stress granules (SGs). SGs form by multivalent interactions between RNAs and RNA-binding proteins and are believed to protect stalled translation initiation complexes from stress-induced degradation. SGs contain hundreds of different mRNAs and proteins, and their assembly and disassembly are tightly controlled by post-translational modifications. The ubiquitin system, which mediates the covalent modification of target proteins with the small protein ubiquitin (‘ubiquitylation’), has been implicated in different aspects of SG metabolism, but specific functions in SG turnover have only recently emerged. Here, we summarize the evidence for the presence of ubiquitylated proteins at SGs, review the functions of different components of the ubiquitin system in SG formation and clearance, and discuss the link between perturbed SG clearance and the pathogenesis of neurodegenerative disorders. We conclude that the ubiquitin system plays an important, medically relevant role in SG biology.

## 1. Introduction

**Figure 1:**
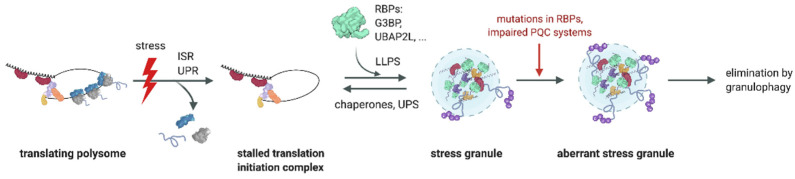
SG assembly and disassembly. Various types of stress induce translational arrest and poly-some disassembly via the integrated stress response (ISR) and unfolded protein response (UPR) pathways. 40S ribosomal subunits, translation initiation factors, mRNPs, and RBPs condensate into SGs via liquid–liquid phase separation (LLPS). RBPs such as G3BP and UBAP2L are central nodes of the protein–RNA network driving LLPS. SGs are disassembled with the help of molecular chaperones and the ubiquitin proteasome system (UPS), allowing translation to recommence. Mutations in RBPs or impairment of protein quality control (PQC) systems mediating SG disassembly promote the transformation into aberrant SGs, accumulating misfolded proteins that are partially modified with ubiquitin chains (purple circles). Aberrant SGs are removed via a selective autophagy pathway termed ‘granulophagy’.

Cells are constantly endangered by various environmental and biotic stresses, such as high temperature, oxidative stress, and viral infections. They adapt to these unfavorable conditions by repressing bulk translation and thereby constraining the damage caused by stress. This is mainly achieved by the induction of the integrated stress response (ISR) and the unfolded protein response (UPR), which converge at the phosphorylation of eukaryotic initiation factor 2 alpha (eIF2α), resulting in translational arrest, polysome disassembly, and the formation of stress granules (SGs) (Figure 1) [1,2,3].

SGs are membrane-less cytoplasmic organelles consisting of messenger ribonucleoprotein complexes (mRNPs), RNA binding proteins (RBPs), translation initiation factors, and numerous additional proteins, including misfolded proteins [4,5,6]. Many SG-associated RBPs, such as G3BP1/2 (henceforth referred to as G3BP), UBAP2L, TIA-1, hnRNPA1, and the m6A-modified mRNA binding proteins YTHDF1/2/3, contain prion-like, low complexity domains and/or intrinsically disordered regions (IDRs). These structural elements promote the formation of SGs by liquid–liquid phase separation (LLPS) and are driven by multiple protein–protein, RNA–protein, and RNA–RNA interactions [7,8,9]. The specific composition of SGs has been found to vary under different stress conditions, for instance, for SGs induced by heat, arsenite, and ER stress [10,11,12,13]. While the physiological function of SGs is insufficiently understood, they are believed to protect mRNAs and stalled translation initiation complexes in order to reduce the cost of de novo protein synthesis after stress release [14,15,16]. Interestingly, it was shown that G3BP mediates the partitioning of transcripts between polysomes and SGs to reprogram translation towards stress adaptation [17]. Beyond their functions in translation control, SGs have emerged as hubs for cellular signaling proteins, thereby preventing or delaying stress-induced cell death as well as viral infections. Upon oxidative stress, the hyperactivation of mTORC1 signaling is counteracted by the recruitment of the regulatory associated protein of mTOR (Raptor) to SGs, resulting in the inhibition of apoptosis [18,19]. Sequestration of the signaling scaffold protein receptor for activated C kinase 1 (RACK1) into SGs inhibits apoptosis by suppressing stress-responsive MAP kinase pathways [20,21]. Upon stress treatment, Ras homolog gene family member A (RhoA) and Rho-associated, coiled-coil-containing protein kinase 1 (ROCK1) are activated and sequestered into SGs to suppress JNK pathway-dependent apoptosis by preventing phosphorylation of JNK-interacting protein 3 [22]. SGs induced by viral infections recruit proteins that control innate immunity, such as dsRNA-dependent protein kinase (PKR), the dsRNA helicase RIG-I, and the cytosolic DNA sensor cGAS, thereby activating them to initiate an antiviral cellular response. Conversely, some viruses undermine SG formation to promote their survival and reproduction [23,24,25,26,27].

When stress conditions subside, SGs must be removed for normal translation to resume. To this end, several types of SGs disassemble with the help of molecular chaperones (Figure 1). Both pharmacological inhibition and siRNA-mediated depletion of HSP70 were found to delay SG removal [28,29]. The small heat shock protein HSPB8 associates with arsenite-induced SGs, prevents the aggregation of misfolded proteins, and recruits BAG3 and HSP70 to promote the removal of misfolded proteins from SGs. Failure in HSPB8–BAG3–HSP70-mediated SG disassembly leads to the accumulation of misfolded proteins inside SGs and to their subsequent transformation into a less dynamic and persistent state [28,29]. Misfolded proteins within such aberrant SGs are believed to be prone to aggregate into fibrils, which is a hallmark in the pathogenesis of a number of neurodegenerative and neuromuscular disorders. Aberrant SGs were shown to be degraded by a p62/SQSTM1-mediated selective autophagy pathway termed ‘granulophagy’ [28,29,30,31,32,33].

SG dynamics are heavily regulated by post-translational modifications (PTMs) such as phosphorylation and methylation, which have been extensively reviewed elsewhere [34,35]. Here, we focus on the covalent modification with the small protein ubiquitin (Ub), referred to as ‘ubiquitylation’. Ubiquitylation classically involves the formation of an isopeptide bond between the C terminus of Ub and lysine residues of target proteins. This ATP-dependent process requires a catalytic cascade of three enzymes, E1 (Ub-activating enzyme), E2 (Ub-conjugating enzyme), and E3 (Ub protein ligase) [36]. Proteins can be modified with one Ub molecule (‘mono-ubiquitylation’) or several individual Ub molecules (‘multi-ubiquitylation’). More frequently, however, the conjugated Ub moiety is in turn ubiquitylated on one of its seven lysine residues or its free amino terminus, resulting in target protein modification with Ub chains of different lengths and linkage types (‘poly-ubiquitylation’). Importantly, the type of Ub modification defines the downstream fate of target proteins [37]. Chains linked via lysine residue 48 of Ub (‘K48 chains’) are prototypical signals for targeting to, and degradation by, the 26S proteasome through pathways collectively known as the ‘Ub proteasome system’ (UPS). The UPS is essential for the elimination of misfolded or otherwise damaged proteins by cellular protein quality control (PQC) mechanisms, as well as for the precisely controlled degradation of transcription factors, cell cycle regulators, and metabolic enzymes. Other types of Ub modifications, such as K63 chains and mono-ubiquitylation, control proteasome-independent processes including autophagy, endolysosomal trafficking, and DNA damage repair. Key to the distinct functions of individual Ub modifications is their specific recognition by effector proteins possessing dedicated Ub-binding domains, which direct the ubiquitylated proteins to different downstream pathways [38,39]. Importantly, Ub modifications can be edited or removed by deubiquitylating enzymes (DUBs), which further increases the plasticity of this highly versatile PTM [40]. Moreover, many Ub-controlled cellular processes require the activity of the abundant ATPase p97 (also known as VCP and Cdc48), which unfolds ubiquitylated proteins to prepare them for their designated downstream pathways [41,42].

In this review, we examine the relationship between SGs and the Ub system. We first discuss the association of Ub conjugates with various types of SGs and then describe how components of the Ub system contribute to both SG assembly and disassembly. Finally, we discuss how malfunction of the Ub system contributes to perturbed SG homeostasis (referred to as ‘granulostasis’), which has been linked to human diseases.

## 2. Presence of Ubiquitin and Ub-like Modifiers at SGs

Various lines of evidence link the Ub system with granulostasis. Treatment of cells with the proteasome inhibitor MG132 induces the formation of SGs, likely by causing the transient overloading of HSP70 chaperones with misfolded proteins [43]. More direct evidence comes from the finding that Ub and various Ub-related proteins associate physically with SGs. The presence of Ub at SGs has been reported by a number of immunofluorescence microscopy studies using different cell lines and SG-inducing conditions [29,44,45,46,47,48,49,50,51,52]. However, the identity of the Ub signals with respect to chain type and target proteins has remained elusive in most of these studies. Recently, two studies even reported that SGs contain free mono-Ub and/or non-conjugated Ub chains [46,52]. By contrast, a recent comparative analysis provided strong evidence that SGs contain Ub conjugates under various stress conditions, albeit with varying frequency [47]. Indeed, SG-associated Ub was detected by linkage-specific anti-Ub antibodies in this and other studies [46,47,48,49], excluding the possibility that it represents either free mono-Ub or mono-ubiquitylated target proteins. Moreover, a recombinant sensor protein binding with high affinity to the free C terminus of mono-Ub and non-conjugated Ub chains did not produce significant signals at SGs, indicating that both are not major SG-associated species [47,53].

Given that SGs contain Ub conjugates, what are the ubiquitylated target proteins? Many SG-inducing conditions such as heat shock and arsenite stress are proteotoxic and lead to the accumulation of defective proteins exposing otherwise buried side chains that could mediate SG association in analogy to the IDRs of core SG proteins. Indeed, misfolded proteins such as defective ribosomal products (DRiPs) and mutant SOD1 were found at SGs induced by MG132, arsenite, and heat stress [28,29,45]. Since these and other misfolded proteins are targets of the UPS, it has been proposed that ubiquitylated proteins at SGs comprise PQC substrates [28,29,31,45]. However, two recent reports indicated that PQC substrates may not be the only ubiquitylated species present at SGs. Ub was also found to associate with light-induced, SG-like granules (‘OptoGranules’) in the absence of exogenous proteotoxic stress [54], suggesting that some non-PQC SG component(s) might be ubiquitylated during SG formation. Indeed, G3BP itself has recently been shown to undergo K63-linked ubiquitylation upon heat stress as a prerequisite for subsequent p97-mediated SG disassembly (see below) [49]. Nevertheless, the identity of most ubiquitylated proteins at SGs is still obscure and requires further investigation.

In addition to known and unknown ubiquitylated target proteins, several Ub-related proteins were found to associate with SGs. These include the E3s TRIM25, Riplet, and MEX3C; the DUBs USP5, USP10, USP13, and OTUD4; the Ub-binding proteins UBAP2L, UBQLN2, and HDAC6; and p97 and the 26S proteasome. Their presence at SGs further underscores the tight links between the Ub system and SG biology. TRIM25, Riplet, and MEX3C ubiquitylate, and thereby activate, RIG-I at virus-induced SGs [55,56], but are apparently not involved in the formation and disassembly of SGs. By contrast, the other aforementioned Ub-related proteins have been directly linked to SG metabolism, and their specific functions are discussed in the next sections.

Besides Ub, the two Ub-like modifiers SUMO and NEDD8 have been implicated in SG biology. Several SUMOylation enzymes, as well as SUMOylated eIF4A, were found to associate with SGs, and pharmacological inhibition of the SUMO E1 enzyme, as well as the depletion of the SUMO-targeted Ub ligase RNF4, caused a significant delay in SG clearance [57,58,59]. However, convincing evidence that SUMO conjugates beyond eIF4A are actually present at SGs is still missing [59]. Instead, it has been suggested that inhibition of the SUMO system affects SG dynamics indirectly by increasing the levels of PQC substrates [59]. Conflicting data exist on the role of NEDD8, with one study demonstrating that NEDDylation of the splicing factor SRSF3 promotes SG assembly [60], and another study concluding that an active NEDDylation system is not required for SG assembly or clearance [52]. Because no clear picture about the functions of SUMO and NEDD8 in SG metabolism has emerged yet, we focus here on the role of the Ub system in SG formation and clearance.

## 3. Role of the Ub System in SG Formation

The potential role of the Ub system in SG formation has been addressed by pharmacological inhibition of the system and by the functional characterization of SG-associated, Ub-related proteins. Broad inhibition of ubiquitylation by the E1 inhibitor TAK-243 does not prevent the formation of SGs induced by arsenite [47,50,52] or heat stress [47,49,50], strongly suggesting that SG formation does not require ongoing ubiquitylation. It should, however, be noted that these results do not formally exclude the possibility that long-lived Ub conjugates formed before the addition of the E1 inhibitor could be involved in SG assembly.

Considering the results obtained with the E1 inhibitor, it may at first glance seem surprising that two DUBs have been reported to play a role in SG formation. USP10 has long been known to associate with SGs induced by various stresses, but conflicting data exist on its role in SG formation. In one study, the overexpression of USP10 prevented the formation of SGs, whereas its depletion affected SG formation very weakly at best [61]. These authors further showed that USP10 competes with Caprin1 for G3BP binding and concluded that USP10 is an inhibitor of SG assembly. By contrast, two other studies found that the depletion or knockout of USP10 significantly reduced SG formation, whereas overexpression of USP10 induced SG formation, indicating that USP10 promotes SG formation [62,63]. Important in the context of this review, however, is the fact that, in both depletion-complementation and overexpression experiments, wild-type and catalytically inactive mutant USP10 had similar effects. These data strongly suggest that the DUB activity of USP10 is dispensable for the regulation of SG formation [62,63]. Interestingly, USP10 possesses an apparently SG-independent function in ribosome-associated quality control, where it antagonizes the ubiquitylation of several subunits of the 40S subunit to prevent its degradation [64,65]. OTUD4 is a member of the ovarian tumor family of DUBs and preferentially cleaves K48 chains [66]. OTUD4 is an RBP, interacts with G3BP in an RNA-dependent manner, and localizes to arsenite- and heat-induced SGs [67]. siRNA-mediated depletion of OTUD4 increased the number of SGs per cell while reducing their size. Thus, OTUD4 appears to be involved in the maturation of SGs, possibly by promoting SG fusion. Similar to USP10, however, wild-type and catalytically inactive mutant OTUD4 rescued the defect with comparable efficiency, suggesting that the DUB activity of OTUD4 is dispensable for SG formation [67]. In summary, the SG-associated DUBs USP10 and OTUD4 appear to affect SG formation by mechanisms that do not involve their catalytic activity. It can, however, not be excluded that USP10 and OTUD4 additionally deubiquitylate some SG-resident proteins to regulate their stability and/or localization to SGs.

In addition to the DUBs USP10 and OTUD4, several Ub-binding proteins have been implicated in SG formation. UBAP2L (Ub-associated protein 2-like) contains several predicted RNA binding regions, a UBA domain predicted to bind to ubiquitylated proteins, and a DUF3697 domain proposed to bind G3BP [68]. UBAP2L was reported to associate with Ub-positive aggregates formed upon proteasome inhibition [69]. Importantly, UBAP2L has been shown to nucleate SGs in the presence of various stresses, such as arsenite, endoplasmic reticulum, and heat stress [10,68,70]. siRNA-mediated depletion or the CRISPR/Cas9-mediated knockout of UBAP2L strongly inhibit SG formation, suggesting that UBAP2L is an essential SG nucleator acting upstream of G3BP [10,70]. While overexpression of full-length UBAP2L in *UBAP2L* KO cells efficiently restored the formation of SGs, overexpression of UBAP2L lacking the UBA domain caused a partial rescue, suggesting that the UBA domain may contribute to SG formation [68,71]. However, it is not known if UBAP2L actually binds to (a) ubiquitylated protein(s) during SG formation. The cytoplasmic deacetylase HDAC6, which mediates the microtubular transport of misfolded ubiquitylated proteins to aggresomes, has been demonstrated to localize to SGs through interaction with G3BP [44]. Both pharmacological and genetic inactivation of HDAC6 were found to impair SG formation [44,51]. Importantly, it was shown that the Ub-binding ZnF-UBP domain of HDAC6 was required for SG formation [44], suggesting that the HDAC6-mediated transport of ubiquitylated proteins is critical for this process. By contrast, in a recent study, deletion of the ZnF-UBP domain of HDAC6 did not prevent the formation of SGs induced by infection with Coxsackievirus A16, but it almost completely abolished the occurrence of poly-Ub-positive SGs [51]. These latter results would be consistent with a scenario in which ubiquitylated proteins are present at SGs, but SG formation does not depend on their presence. UBQLN2 (ubiquilin-2) contains an N-terminal Ub-like domain mediating proteasome binding and a C-terminal UBA domain. It has been characterized as a shuttle protein, delivering substrates to the 26S proteasome, but is also involved in autophagy [72,73]. UBQLN2 associates with SGs induced by various stresses and negatively regulates SG formation [74,75]. While the depletion of UBQLN2 accelerates the formation and increases the size of SGs, its overexpression impairs SG formation [74,75]. Interestingly, UBQLN2 was found to undergo LLPS in vitro [76,77], a process that was abolished by Ub binding, possibly through a reduction in the multivalent interactions of UBQLN2 [76]. This led to the proposal that UBQLN2 might modulate the composition of SGs. According to this model, UBQLN2 associates with SGs through LLPS. Upon binding to ubiquitylated proteins at SGs, UBQLN2 reverts to the dilute phase, dissociates from the SGs, and thereby extracts ubiquitylated proteins from the SG for subsequent degradation [76]. While the details of this model await experimental verification, it should be noted that the increased SG formation observed upon UBQLN2 depletion might also result from elevated levels of PQC substrates due to inefficient proteasomal delivery. Taken together, the accumulated evidence strongly suggests that the Ub system is not critically required for the formation of SGs, but it leaves open the possibility that the presence of ubiquitylated proteins, DUBs, and Ub-binding proteins at SGs may modulate the dynamics of SG formation.

## 4. Role of the Ub System in SG Clearance

While there is currently little evidence for a critical role of the Ub system in SG formation, its role in SG clearance is well-supported both by experiments using pharmacological inhibitors of various enzymes of the Ub system and by a number of mechanistic studies. Several recent reports have described the effect of the E1 inhibitor TAK-243 on SG clearance. In one study, cells pretreated with TAK-243 were subjected to arsenite stress, followed by the washout of arsenite. Both control and TAK-243-treated cells showed a similar efficiency of SG clearance, suggesting that an active Ub system is not required for clearance [52]. By contrast, a study replicating this experiment found a significant SG clearance defect after TAK-243 pretreatment [47]. To exclude indirect effects of the inhibitor pretreatment on SG composition and dynamics, these authors also analyzed the effect of acute E1 inhibition limited to the recovery phase and found a significant delay in the clearance of both arsenite- and heat-induced SGs [47]. Finally, a third study found that cells pretreated with TAK-243 could not efficiently clear heat-induced SGs, whereas the clearance of arsenite-induced SGs was not significantly affected [50]. Thus, while there is agreement on a requirement for an active Ub system for the efficient clearance of heat-induced SGs, conflicting data exist regarding the clearance of arsenite-induced SGs. Even though cell line-specific differences may contribute to the discrepancies, additional work is needed to clarify this point. In addition to the E1 inhibitor TAK-243, the effects of various other pharmacological inhibitors of the Ub system on SG clearance have been examined. The presence of the proteasome inhibitor bortezomib and of the DUB inhibitors PR-619 (a general DUB inhibitor) and b-AP15 (mainly targeting proteasome-associated DUBs) during stress recovery strongly impaired the clearance of arsenite-induced SGs and moderately delayed the clearance of heat-induced SGs [47]. Moreover, several studies showed that inhibition of the ATPase p97 causes a significant delay in the clearance of both arsenite- and heat-induced SGs [30,47,49]. Together, these reports strongly suggest that the turnover of ubiquitylated proteins is required for the efficient clearance of arsenite- and heat-induced SGs.

This conclusion is further supported by functional studies of enzymes of the Ub system that were found to associate with SGs. The DUBs USP5 and USP13 were shown to be present at SGs induced by heat stress or by the combined treatment with puromycin and the HSP70 inhibitor VER-155008 [46]. siRNA-mediated depletion of USP5 and USP13 increased the association of Ub and impaired the clearance of heat-induced SGs. This defect was rescued by the reintroduction of wild-type, but not catalytically inactive, USP5 and USP13, demonstrating that the DUB activities of both DUBs are necessary for efficient SG clearance of heat-induced SGs [46]. Interestingly, USP5 (but not USP13) preferentially cleaves non-conjugated Ub chains, suggesting that heat-induced SGs contain Ub conjugates and free Ub chains that both need to be hydrolyzed for efficient SG clearance to occur.

**Figure 2:**
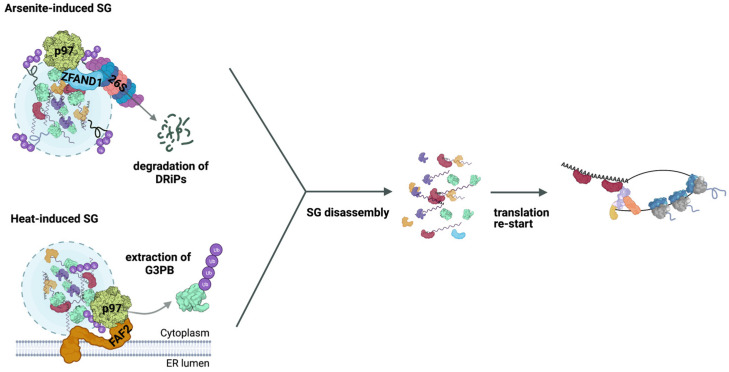
SG disassembly by p97 and its cofactors. Top: The p97 cofactor ZFAND1 (blue) associates with arsenite-induced SGs and recruits both p97 (yellow) and the 26S proteasome, promoting SG disassembly by removing ubiquitylated (purple), presumably misfolded proteins, such as DRiPs. Bottom: The ER membrane-localized p97 cofactor FAF2 (orange) recruits p97 to heat-induced SGs, where it promotes disassembly by removing ubiquitylated G3BP. After SG disassembly, productive translation initiation complexes can form to restart translation.

The ATPase p97 has been shown to associate with SGs under various stress conditions [30,45,47,49,78], and there is clear evidence for its critical role in SG clearance. In line with the effects of pharmacological p97 inhibitors mentioned above, the siRNA-mediated depletion of p97 was found to interfere with efficient SG clearance [30,49]. The activity of p97 at SGs is regulated by the autophagy-inducing kinases ULK1/2 [78]. ULK1/2 localize to SGs and phosphorylate p97 on residues Ser13, Ser282, and Thr761 to stimulate its ATPase activity. Pharmacological inhibition or siRNA-mediated depletion of ULK1/2 impaired the disassembly of heat- and arsenite-induced SGs in U2OS and C2C12 cells. Conversely, the expression of a phosphorylation-mimicking p97 variant abolished the ULK1/2 dependency, demonstrating that ULK1/2-dependent phosphorylation of p97 is critical for normal SG clearance [78]. Importantly, mutations in the *VCP* gene encoding p97 cause the degenerative human disease multisystem proteinopathy 1 (MSP1; also known as inclusion body myopathy associated with Paget’s disease of bone, frontotemporal dementia, and amyotrophic lateral sclerosis, IBMPFD/ALS) [79,80], and pathogenic p97 variants have been reported to affect SG clearance. Ectopic expression of two disease-causing p97 variants (R155H and A232E) significantly delayed the clearance of SGs induced by heat or arsenite in different cell lines [45,49,78,81], thereby linking perturbed granulostasis to MSP1. The requirement for active p97 at SGs for their normal clearance is further supported by the identification of two regulatory cofactors that recruit p97 to SGs. ZFAND1 was found to associate with arsenite-induced SGs and to recruit both p97 and the 26S proteasome, enabling them to remove DRiPs and presumably additional ubiquitylated proteins from the SGs (Figure 2) [45]. In the absence of ZFAND1, p97 and the 26S proteasome fail to localize to arsenite-induced SGs, resulting in the accumulation of DRiPs at SGs, delayed SG clearance, and the transformation of normal SGs into aberrant SGs [45]. The p97 cofactor FAF2 (also known as UBXD8), an integral membrane protein of the endoplasmic reticulum (ER), was shown to recruit p97 to heat-induced SGs [49]. Intriguingly, Gwon et al. found that G3BP is marked with K63 chains upon heat stress, and that its p97- and FAF2-dependent extraction from SGs is required for the efficient clearance of heat-induced SGs (Figure 2) [49].

Interestingly, the cofactor dependency of p97 is strictly SG-specific: Whereas ZFAND1 is required for the clearance of arsenite- but not heat-induced SGs, the opposite is true for FAF2, indicating that significant differences exist in the composition and, perhaps, the localization of these two paradigmatic types of SGs. While G3BP is the first defined and critical ubiquitylated substrate at SGs, its fate following p97-mediated extraction is not known. On the one hand, K63 chains do not usually target proteins for proteasomal degradation, suggesting that G3BP could be either degraded via autophagy or other lysosomal pathways, or simply recycled to the cytosol after removal of the Ub chain by some DUB. On the other hand, FAF2 has so far been implicated in p97- and proteasome-dependent proteolysis, most prominently in the ER-associated degradation pathway [82,83]. Accordingly, it has been suggested that, upon heat shock, p97 extracts G3BP that is misfolded and co-aggregated with DRiPs at SGs for proteasomal degradation [84]. While this elimination of a PQC substrate would be in line with known functions of p97 and FAF2, it would typically require modification with a K48 chain. Clearly, additional experiments are required to fully understand the Ub-dependent removal of G3BP from heat-induced SGs. Notably, p97 possesses additional, still poorly characterized functions during the autophagic elimination of aberrant SGs that escaped normal clearance [30], further underscoring its key role in granulostasis.

The 26S proteasome was shown to associate with arsenite- and heat-induced SGs, and its inhibition by bortezomib impairs the clearance of both types of SGs [45,47]. DRiPs are short-lived substrates of the UPS that are marked primarily with K48 chains, and are rapidly degraded by the 26S proteasome with the help of p97 [85,86]. DRiPs have been reported to accumulate at SGs, both in the presence of bortezomib and upon depletion of ZFAND1 [28,45]. Together, these findings strongly suggest that the 26S proteasome removes DRiPs and potentially other ubiquitylated PQC substrates from SGs (Figure 2), thereby promoting efficient SG disassembly and preventing the formation of aberrant SGs [45].

## 5. Perturbed Granulostasis Is Linked to Neurodegenerative Disorders

Impaired SG dynamics have been implicated in the pathogenesis of neurodegenerative disorders, including amyotrophic lateral sclerosis (ALS), frontotemporal dementia (FTD), multisystem proteinopathy (MSP), Alzheimer’s disease, and Parkinson’s disease [87,88], which are characterized by toxic protein aggregation in neurons and progressive neuronal loss [89,90]. It was proposed that aberrant SGs, which are less dynamic than normal SGs and contain misfolded, aggregation-prone proteins that tend to fibrillize and undergo liquid-to-solid transitions, are seeds for the formation of pathogenic aggregates [31,88,91,92]. Moreover, data from *C. elegans* indicate that aberrant SGs become highly abundant in older animals [93], linking impaired granulostasis with aging. Aberrant SGs can develop either due to an altered SG composition enriched in PQC substrates and other aggregation-prone proteins, or upon the impairment of proteostasis factors promoting normal SG clearance [28,29,45]. In accordance with the first scenario, several SG-resident RBPs, including TDP-43, FUS, hnRNPA1, and TIA-1, were found to carry pathogenic mutations in the tissues of affected individuals, which alter their propensity to associate with SGs [87,88,94]. TDP-43 (Tar DNA-binding protein-43) is frequently present in ubiquitylated inclusions in brain/muscle sections from patients with ALS and/or FTD [80,95]. Disease-linked mutations in TDP-43 increase its nuclear export and association with SGs, thereby presumably inducing their transition into aberrant SGs [96,97,98]. The SG-associated protein FUS (fused in sarcoma) is exported from the nucleus to the cytoplasm under stress conditions and associates with SGs. Several disease-linked mutations in FUS cause its cytoplasmic mislocalization and increase its association with SGs [87,99,100,101]. At SGs, mutant FUS may then aggregate into cross-beta fibril structures, thereby promoting a liquid-to-solid phase transition of SGs [31,94,100].

Another source of aggregation-prone proteins at SGs are misfolded proteins that evade or overwhelm cellular PQC systems. Hexanucleotide repeat expansions in the *C9ORF72* gene are the most frequent genetic alteration found in ALS/FTD patients [102,103]. Importantly, they were shown to cause the accumulation of SGs in an induced pluripotent stem cell model of ALS/FTD in the absence of exogenous stress [104]. Among other potentially pathogenic effects, the hexanucleotide repeat expansions lead to the non-canonical translation of dipeptide repeat proteins, which have a high propensity to undergo LLPs in vitro and, at least upon ectopic overexpression, accumulate at SGs in cells, thus perturbing SG dynamics [31,103,105,106]. Similar to dipeptide repeat proteins, misfolding-prone variants of proteins that are not normally present at SGs can change the properties of SGs. For instance, an ALS-linked, misfolding-prone variant of SOD1 (superoxide dismutase 1) was observed to accumulate at heat-induced SGs, whereas wild-type SOD1 was absent [29]. At SGs, the mutant SOD1 exhibited reduced mobility, probably due to aggregation. Mutant SOD1-positive SGs in turn became less dynamic over time and showed reduced fusion and fission events. Importantly, these mutant SOD1-positive SGs possessed higher levels of chaperones, Ub, and p97, suggesting that cellular PQC systems, including the Ub system, are engaged in preventing the formation of aberrant SGs by removing misfolded proteins from SGs [29]. Similarly, the accumulation of DRiPs at SGs was found to correlate with an increase in persistent, bona fide aberrant SGs [28,45].

Besides the enrichment of aggregation-prone proteins at SGs, the mutational impairment of several proteostasis factors promoting normal SG dynamics has been linked to neurodegenerative disorders [31,79,107,108,109]. These include the SG assembly limiter UBQLN2 mutated in ALS/FTD [74,75,110,111]; HSPB8 and BAG3 from the SG-disassembling chaperone axis HSPB8–BAG3–HSP70 mutated in Charcot–Marie–Tooth disease, distal hereditary motor neuropathy, and distal myopathy [107,108,112]; the granulophagy adaptor p62/SQSTM1 mutated in ALS/FTD and Alzheimer’s disease [113,114,115]; and p97 mutated in MSP1 [79,109]. Taken together, these findings strongly implicate the cellular PQC machinery, including several of the Ub system components discussed in this review, in normal SG physiology and its pathological aberration in neurodegenerative disorders.

## 6. Conclusions and Perspective

SG metabolism is governed by intermolecular interactions driving LLPS and is controlled on multiple levels by PTMs. This review focuses on the role of the Ub system in SG dynamics. There is now clear evidence that SGs contain Ub conjugates of different linkage types, and that inhibition of various enzymes of the Ub system interferes with normal SG dynamics. While an active Ub system is probably not required for (but could still be involved in) SG assembly, the removal of ubiquitylated proteins from SGs mediated by p97, the 26S proteasome, and DUBs is critical for the efficient disassembly of heat- and arsenite-induced SGs. Failure to remove ubiquitylated proteins from SGs promotes the formation of aberrant SGs, which are strongly implicated in neurodegenerative disorders such as ALS and FTD. Thus, the Ub system has emerged as an important, medically relevant player in SG biology. A better mechanistic understanding of Ub-dependent processes in SG clearance is likely to shed new light on the pathogenesis of neurodegenerative disorders and may help to devise new therapeutic strategies to tackle them. Nevertheless, there are many open questions that need to be addressed by future research. First and foremost, while G3BP is so far the only identified ubiquitylated SG component critical for SG disassembly, its ubiquitylation is specifically observed upon heat stress. What other ubiquitylated target proteins are critical for the turnover of heat-induced and other types of SGs? Identifying the ubiquitylomes of different types of SGs should provide insights into their differential composition and requirements for clearance. Second, G3BP was found to be modified with K63 chains, but K48 and K29 chains are also present at SGs. What is the fate of G3BP and other unknown ubiquitylated proteins following their removal from SGs? Are they degraded in the context of cellular PQC networks, or does their ubiquitylation (and de-ubiquitylation) serve regulatory purposes? Third, which E3 ligases and DUBs (beyond USP5 and USP13) control the ubiquitylation of SG proteins? Their identification will be important for devising more specific experimental approaches to study the functional consequences of protein ubiquitylation at SGs. Finally, SG biology has predominantly been studied in cellular models under harsh stress conditions. While the depicted links between aberrant SGs and neurodegenerative disorders are highly suggestive, they need to be scrutinized in appropriate animal models. In a first step towards this aim, a transgenic ALS mouse model was recently used to demonstrate that the expression of mutant *FUS* also causes delayed SG clearance in vivo [116]. Further work is needed to explore whether aberrant SGs actually form in vivo, if they are indeed seeds for pathogenic protein aggregates, and if and how a compromised Ub system affects their clearance.
